# Recent Developments in the Field of Optical Immunosensors Focusing on a Label-Free, White Light Reflectance Spectroscopy-Based Immunosensing Platform

**DOI:** 10.3390/s22145114

**Published:** 2022-07-07

**Authors:** Chrysoula-Evangelia Karachaliou, Georgios Koukouvinos, Dimitrios Goustouridis, Ioannis Raptis, Sotirios Kakabakos, Evangelia Livaniou, Panagiota Petrou

**Affiliations:** 1 Immunopeptide Chemistry Lab, Institute of Nuclear & Radiological Sciences & Technology, Energy & Safety, National Centre for Scientific Research ‘‘Demokritos”, P.O. Box 60037, 153 10 Agia Paraskevi, Greece; 2 Immunoassay/Immunosensors Lab, Institute of Nuclear & Radiological Sciences & Technology, Energy & Safety, National Centre for Scientific Research ‘‘Demokritos”, P.O. Box 60037, 153 10 Agia Paraskevi, Greece; geokoukoubinos@yahoo.gr (G.K.); skakab@rrp.demokritos.gr (S.K.); ypetrou@rrp.demokritos.gr (P.P.); 3 ThetaMetrisis S.A., 121 32 Athens, Greece; dgoustouridis@thetametrisis.com (D.G.); raptis@thetametrisis.com (I.R.); 4 Department of Electrical & Electronics Engineering, University of West Attica, 122 44 Athens, Greece; 5 Institute of Nanoscience and Nanotechnology, National Centre for Scientific Research ‘‘Demokritos”, P.O. Box 60037, 153 10 Agia Paraskevi, Greece

**Keywords:** analyte, antibody, optical immunosensors, labeled and label-free immunosensor platforms, white light reflectance spectroscopy, bioanalytical applications

## Abstract

Optical immunosensors represent a research field of continuously increasing interest due to their unique features, which can mainly be attributed to the high-affinity and specific antibodies they use as biorecognition elements, combined with the advantageous characteristics of the optical transducing systems these sensors employ. The present work describes new developments in the field, focusing on recent bioanalytical applications (2021–2022) of labeled and label-free optical immunosensors. Special attention is paid to a specific immunosensing platform based on White Light Reflectance Spectroscopy, in which our labs have gained specific expertise; this platform is presented in detail so as to include developments, improvements, and bioanalytical applications since the mid-2000s. Perspectives on the field are been briefly discussed as well, highlighting the potential of optical immunosensors to eventually reach the state of a reliable, highly versatile, and widely applicable analytical tool suitable for use at the Point-of-Care.

## 1. Introduction

Immunosensors can be described as devices capable of detecting/quantifying certain analytes in various samples. As the “immuno”-part of the term denotes, immunosensors are based on specific antibodies that recognize the analyte and allow its consequent assay; on the other hand, they are based on appropriate transducers that can detect/transform the assay signal and reliably “translate” it into detection/quantification of the analyte present in the sample of interest. 

Depending on the transducer systems they employ, immunosensors can be further divided into groups, such as electrochemical, piezoelectric, or optical ones [1,2]. Among the above-mentioned groups, optical immunosensors have proven very popular; while quite similar in principle, for a number of reasons they represent the next technological step compared to conventional immunoassays, the vast majority of which are based on optical signal/detection. More specifically, optical immunosensors offer short analysis times and high sample throughput, low interference caused by the sample matrix, and potential for multiplexed analysis and reusage, as well as automation and integration with microfluidic systems into miniaturized instruments for Point-of-Care applications [1,2].

### Labeled vs. Label-Free Optical Immunosensors

Early optical immunosensors used specific labels that were linked to a suitable assay-biomolecule, enabling detection of the analyte–antibody interactions and quantification of the analyte based on appropriate characteristics of the optical signal these labels emit. The most widely used labels are fluorescent, ranging from well-known classical dyes such as Cy5 [3] or Dy654 [4] to specially-prepared quantum dots [5]. Based on various criteria, fluorescent immunosensors can be further subdivided, e.g., into sensors based on fluorescence quenching, which mostly depends on binding or removal of the analyte to/from the specific antibody [5,6,7]. Among the latter, immunosensors employing so-called quenchbodies (Q-bodies) may be considered as a separate sub-group of sensors, which are also based on the principle of fluorescence quenching. Q-bodies are antibody-variable regions labeled with one (or two) fluorescent dye(s) near the antigen-binding site, the fluorescence intensity of which differs between the analyte-free and analyte-bound state [8,9,10,11]. According to the relevant literature, other principles of fluorescence-based detection methods deserve special attention; e.g., two-photon fluorescence is considered a field of particular interest [12], with many potential applications in bioassays/microscopy [13,14].

Despite the wide application of labeled immunosensors, many research efforts have focused on label-free approaches [15,16,17]. Label-free optical immunosensors can create a signal directly upon binding of the analyte to the specific antibody that is usually immobilized onto the sensor’s surface, enabling real-time monitoring of the immunochemical reaction and development of fast bioanalytical assays.

The majority of label-free optical immunosensors rely on optical signals measured by refractometry or reflectometry in the UV-Vis spectrum [18]. Nowadays, the most widely-applied approaches toward development of label-free optical sensors are based on refractometric methods, in which the change in the refractive index of the biolayer formed on the transducer surface is of special importance, as it influences the transmission of light through the transducer. These methods include mainly surface plasmon resonance (SPR), grating couplers, resonant mirrors, Mach–Zehnder and Young interferometers, and Bragg gratings [18,19]. It is worth mentioning that there are several commercially available instruments based on SPR sensing, which can be employed for routine analysis; SPR biosensors [20,21] may, therefore, be considered of particular interest for bioanalytical applications. On the other hand, reflectometry has not yet been applied as widely as refractometry. Reflectometry focuses on detecting changes in intensity or the phase of light due to changes in the physical thickness of the layer formed by the interacting biomolecules. As originally described in early publications [22,23], white light is reflected at the interphases of the biolayer to create an interference spectrum, which shifts to higher wavelengths as the optical thickness changes; this “reflectometric interference spectroscopy” method has evolved over the years and has been transformed to several similar-in-principle implementations with various applications, including bioanalytical ones. Among these bioanalytical applications, a recent one refers to the determination of the bacterial surface S-layer protein (SLP) by means of an anti-SLP monoclonal antibody immobilized (through bacterial protein A) onto an optically-sensitive TiO_2_-coated porous silica surface [24]. Similarly, recent articles based on reflectometric interference spectroscopy have reported the direct measurement of analytes in complex samples, such as immunoglobulin G (IgG) in whole human blood, by employing silica colloidal crystal film [25,26] as an optical-sensitive platform and protein A as the IgG-binder instead of a specific antibody [27], or more recently, the measurement of IgG in milk following a similar-in-principle strategy [28]. During the last decade, a special type of biosensors based on reflectometric interference spectroscopy, known as Interferometric Reflectance Imaging Sensors (IRIS) [29], have been developed and applied to immunoanalysis of biomolecules, such as beta-amyloid 1-42 in artificial cerebrospinal fluid [30], beta-lactoglobulin and human IgE in unprocessed human serum and whole blood [31], interleukin-6 [32], and intact virions of vesicular stomatitis virus [33]. In addition, IgG has been recently determined in aqueous solutions with an IRIS sensor employing protein A as a specific binder instead of an antibody [34]; moreover, IRIS sensors have been applied to studies of antigen–antibody interactions [35], including determination of affinity constants [36] and binding kinetics [37].

White-light reflectance spectroscopy (WLRS) immunosensors may be considered as a special type of label-free optical immunosensors based on reflectometric interference spectroscopy. Our research group has developed a WLRS-based biosensing platform [38]; initially introduced for conducting kinetic studies of interactions between biomolecules [38] or determining the thickness of polymeric layers [39,40], the WLRS system has several bioanalytical applications nowadays, as will be presented in more detail below.

## 2. Newest Developments in Optical Immunosensors: Bioanalytical Applications

Several recent review articles (2021–2022) refer to optical immunosensors, often among other topics of interest in the field of biosensing. Thus, some of these articles describe optical immunosensors that have been developed for various (groups of) biomolecules [1,41,42], such as COVID-19-related biomarkers [43,44,45,46]. Immunosensing based on optical fiber technology [47,48] and application of spectroscopic ellipsometry to immunosensing [49] have been presented in recent reviews. Multiplexed optical immunosensors that are capable of simultaneously detecting/quantifying more than one analyte of diagnostic interest in the same sample and can be used for Point-of-Care applications have been recently presented as well [2,50]. Surface-Enhanced Raman Spectroscopy lateral flow immunoassays (SERS-based LFIAs) performed on properly functionalized membranes, which may themselves be considered as a type of optical immunosensor, have been described in a review article presenting Raman Scattering-based biosensors [51]. Optical immunosensors employing appropriate dendrimers [52] or various nanomaterials [53] as a means of improving sensing efficiency/sensitivity have been presented in recent reviews.

Most of the latest reports (2021–2022) regarding bioanalytical applications of both labeled and label-free optical immunosensors are summarized in Table 1. Starting with the former group, a simple yet highly useful screening assay for methicillin-resistant *Staphylococcus aureus* that may be considered as a type of colorimetric immunosensor was described; the assay was based on a pair of antibodies against penicillin binding protein 2a (anti-PBP 2a) and employed capture antibodies immobilized on activated cotton swabs along with detection antibodies immobilized on colored polymeric nanoparticles [54]. Moreover, a lately reported immunochromatographic strip (lateral flow immunoassay) for zearalenone can be considered as a type of dual colorimetric/fluorescent sensor; the key reagent of the strip consisted of *Staphylococcus aureus* biosynthesized polymer dots that could generate a colorimetric/fluorescent signal, onto which anti-zearalenone antibodies were coupled through their Fc region [55]. In addition, a fluorescence immunosensor was reported in [56] based on graphene quantum dots functionalized through covalent linking with an anti-neuron-specific enolase antibody (energy donor) along with a hybrid of Ti_3_C_2_-MXene nanosheets and silver nanoparticles (energy acceptor), which exhibited high quenching and energy-transfer efficiency. MXene is a new material with the general formula of M_n+1_X_n_T_x_, where M represents a transition metal, X can be nitrogen and/or carbon, and T_x_ stands for denoting surface functionalization [56]. Moreover, a fluorescence immunosensor based on the optical properties of carbon dots and the well-known metal-enhanced fluorescence effect of silver nanoparticles, both covalently “decorated” with anti-analyte antibodies, was reported for human epididymis protein 4 [57], while a strip fluorescence immunosensor was reported for aflatoxin B1 based on anti-aflatoxin B1 antibodies covalently coupled to quantum dot nanobeads as fluorescent label [58]. In addition, a fluorescence immunosensor for *Salmonella typhi VI* antigen was described based on iron porphyrin-like graphene quantum dots (Fe–N–GQDs) as a novel fluorescent label on which the anti-analyte antibody was covalently immobilized [59], while a fluorescence immunosensor for *Escherichia coli* O157 was reported which employed cell-based (i.e., inactivated *Staphylococcus aureus*-based) fluorescent microspheres labeled with carbon dots and loaded with monoclonal antibodies against *E. coli* O157 through the protein A present on the surface of the inactivated cells [60]. A novel fluorescent immunosensor for microcystin-LR (a toxin produced by *Cyanobacteria*) was reported which employed a Cy5.5-labeled anti-analyte antibody in an elegantly designed light-sheet skew rays-enhanced U-shaped fiber-optic evanescent wave immunosensing platform [61]. In a recently published article, an immunosensor based on an antibody-analyte-aptamer structure and a CRISPR/Cas12a fluorescence detection system was developed on a glass fiber and applied to the detection of small proteins such as INF-γ and insulin [62]. A phosphorescence-based displacement assay was designed for small molecules, especially estrone (E1) and estradiol (E2); phosphorescent labels based on palladium tetrabenzoporphyrin were conjugated to E1 and E2 directly or through a linker moiety, and the labeled molecules thus formed were used in a competitive-type assay for E1 and E2 and measured with an optical device developed for that purpose; the same approach could be used for other small molecules of interest [63]. On the other hand, a portable, fiber-based chemiluminescence immunosensor was developed for methylamphetamine, which was based on biotinylated anti-analyte antibodies along with streptavidin-biotinylated HRP nanocomposites and a chemiluminescence-emitting HRP substrate [64]. A dual chemiluminescence/colorimetric lateral flow immunoassay (LFIA) sensor was described for detecting immunoglobulin A (IgA) against SARS-CoV-2 proteins in serum and saliva of patients with COVID-19. The sensor employed a recombinant nucleocapsid antigen, which specifically bound the anti-SARS-CoV-2 antibodies, and anti-human IgA antibody labeled with either gold nanoparticles or HRP; detection was achieved either through a smartphone camera-based device measuring the color signal emitted by nanogold-labeled anti-human IgA or by means of a contact imaging portable device based on cooled CCD that could measure the chemiluminescence emitted by HRP-labeled anti-human IgA upon reaction with a H_2_O_2_/luminol/enhancer substrate [65].

Many of the optical immunosensors included in Table 1 are label-free, and a brief presentation of their basic principle/reagents has been considered of interest. Thus, a label-free sensor was reported which employed dynamic light scattering to detect the binding of anti-SARS-CoV-2 spike glycoprotein antibodies conjugated with gold nanoparticles to spike glycoprotein through the increase in the conjugates’ size [66]. Moreover, a photoluminescence label-free immunosensor for aflatoxin B1 was developed and integrated with a microfluidic cell. The sensor was based on polyacrylonitrile/zinc oxide (PAN/ZnO) nanofibers, which were suitably treated and then loaded with an anti-analyte antibody; the photoluminescence of PAN/ZnO/antibody nanocomposites changed upon binding of aflatoxin B1 in a concentration-dependent manner [67]. A long-period fiber grating (LPFG) immunosensor that employed an avian antibody (IgY) recognizing *Staphylococcus aureus* was reported; upon bacteria–antibody interaction, an increase in the bioconjugate thickness and density was created and was tracked as a change of resonance wavelength in the LPFG transmission spectrum [68]. An all-fiber-optic immunosensor based on elliptical core helical intermediate fiber grating (E-HIPFG) was developed and evaluated through the detection of human IgG after functionalizing the sensor surface with a goat anti-human IgG antibody [69]. Two immunosensors based on specific antibodies against the Cor a 14-hazelnut allergen and electrochemical or SPR-based optical detection were reported and proposed as a platform for hazelnut allergen analysis in food; mammalian IgG and avian IgY anti-analyte antibodies were immobilized on the gold-coated sensors’ surface, with the latter leading to a better outcome regarding mainly assay specificity [70]. Moreover, an immunosensor for COVID-19 diagnosis was reported based on antibodies against the SARS-CoV-2 nucleocapsid protein; the capture antibodies were conjugated to light scattering particles, while the detection ones were immobilized on an optical waveguide film [71]. In addition, an immunosensor for collagen I was described which employed a half-reduced monoclonal antibody, i.e., an anti-collagen I antibody the intramolecular S-S bridges of which were enzymatically reduced; subsequent covalent immobilization of the so-treated antibody on a monolayer of self-assembled gold nanoparticles led to a properly oriented and fully-functional antibody layer. Then, either electrochemical impedance spectroscopy or SPR were combined with the antibody–gold nanoparticles and the relevant immunosensors were developed [72]. A SPR immunosensor for programmed death ligand 1 (PD-L1) was described based on both an aptamer conjugated to magnetite nanorods containing ordered mesocages and silver nanoclusters (MNOM@AgNPs) and an anti-analyte antibody immobilized on the surface of a gold chip properly treated with *para*-sulfonatocalix[4]arene, thus leading to highly specific recognition of PD-L1 [73]. A SPR immunosensor for cortisol was reported which employed an unclad plastic optical fiber coated with a gold/palladium alloy on which an anti-analyte antibody was covalently linked [74]. A recently published paper has described a SPR-immunosensor for ricin and abrin; the sensor uses monoclonal antibodies for the biotoxins, which have been immobilized onto the SPR-chip surface through protein G [75]. Another recently published paper has reported a SPR-immunosensor with a highly enhanced signal for the detection of CD5 cancer biomarkers; the sensor employed capture anti-CD5 antibodies immobilized on the gold surface of the SPR-disc along with detection anti-CD5 antibodies coupled onto gold-coated magnetic nanoparticles [76]. A SERS immunosensor employing 4-mercaptobenzonitrile as a Raman probe was developed for imidaclopir; the sensor was based on Fe_3_O_4_ magnetic nanoparticles coated with an anti-imidaclopir antibody along with bimetallic nanocuboid particles, containing gold in the core and silver in the shell and linked with the antigen and 4-mercaptobenzonitrile [77]. A harmonic microfiber Bragg grating (H-mFBG) immunosensor for cardiac troponin I was reported based on anti-analyte antibody immobilized onto the fiber, and was designed to operate also in vivo [78]. Novel immunosensors based on optical waveguide light mode spectroscopy (OWLS) were developed and applied to the detection of *Fusarium* mycotoxin zearalenone; both immobilized antibody and immobilized antigen assay formats were set up, with the immunoreagents’ immobilization being performed on epoxy-, amino-, or carboxy-functionalized sensing surfaces [79]. A micro-sized non-spectroscopic optical reflector gadget was fabricated that was based on anti-analyte antibodies conjugated to retroreflective particles; the gadget was integrated with a commercial smartphone and applied to the detection of the creatine kinase-myocardial band [80]. Moreover, a Fabry-Pérot interferometric surface stress sensor was developed and applied to detection of prostate-specific antigen (PSA); the sensor employed anti-analyte antibodies covalently immobilized on a deformable biomembrane constructed on a parylene-C nanosheet support/silicon substrate, and the reflection spectra shifts created upon antibody-analyte binding due to membrane deformation were recorded [81]. Moreover, a planar waveguide immunosensor for zearalenone was described: the waveguide was composed of a thin silicon nitride layer between two thicker silicon dioxide layers, and the sensor worked as a polarization interferometer; the anti-analyte antibody was immobilized on the waveguide via a polyelectrolyte layer on which protein A was adsorbed [82]. In a very recent article, an optical immunosensor for COVID-19 diagnosis was described based on a new polymer-type imprinted photonic crystal film (IPCF) that diffracts light in an angle-dependent way; diffraction intensity decreases when a biomolecule, e.g., a specific antibody, is adsorbed on the film surface, and continues to decrease upon immunoadsorption of the analyte. The sensor employed an antibody against the SARS-CoV-2 spike protein immobilized on the IPCF surface, through which spike proteins present in artificial saliva could be detected with the aid of a smartphone-equipped optical setup [83]. In another recently published article, an optical immunosensor based on total internal reflection ellipsometry was reported; the sensor was applied to the detection and study of polyclonal antibodies circulating in the serum of human individuals after vaccination against COVID-19, which could recognize the spike proteins of three SARS-CoV-2 variants [84]. Finally, four immunosensors that relied on WLRS were reported [85,86,87,88]; these immunosensors will be presented in detail below along with further information concerning the WLRS immunosensing platform.

Bioanalytical applications of optical immunosensors reported in the last couple of years (2021–2022) cover a wide range of areas, from disease diagnosis to food analysis (Table 1). Thus, several of the relevant articles present new optical immunosensors for the detection/quantification of proteins of the SARS-CoV-2 virus or human antibodies developed against them [65,66,71,84]. Moreover, due to the COVID-19 pandemic outbreak and the consequent high interest in new tools for COVID-19 diagnosis, several recent review articles have presented, among other analytical approaches, research efforts toward development of optical immunosensors associated with COVID-19 [43,44,45,46,83]. Several optical immunosensors have been developed for the detection of whole bacteria, such as *Staphylococcus aureus* [68], especially methicillin-resistant *Staphylococcus aureus* [54], *Salmonella typhi VI* [59], *Escherichia coli* O157 [60], and *Salmonella typhimurium* [88]. Other optical immunosensors have been applied to and/or evaluated for the detection/quantification of specific disease biomarkers, such as neuron-specific enolase [56], myocardial creatine-kinase [80], prostate-specific antigen [81], human epididymis protein 4 [57], programmed death ligand 1 [73], cardiac troponin I [78], and C-reactive protein [87]. Another group includes optical immunosensors that have been applied to and/or evaluated through the detection/quantification of basic and important biomolecules, such as human INF-γ or insulin [62], immunoglobulin G [69], CD5 [76], collagen I [72], cortisol [74], and estrone and estradiol [63]. On the other hand, other optical immunosensors have been applied to the detection/quantification of exogenous substances, especially natural or synthetic toxic compounds, such as methylamphetamine [64], ochratoxin A [85], aflatoxin B1 [58,67], microcystin-LR [61], zearalenone [55,79,82], hazelnut Cor a14 allergen [70], imidacloprid [77], carbendazim [86], and ricin and abrin [75].

## 3. WLRS-Based Optical Immunosensors

As already mentioned, WLRS-sensors are a special type of label-free optical sensors. A review article concerning WLRS-based biosensing platforms was published by our group few years ago, providing information on both the operating principles and specific applications in various areas [89].

**Figure 1:**
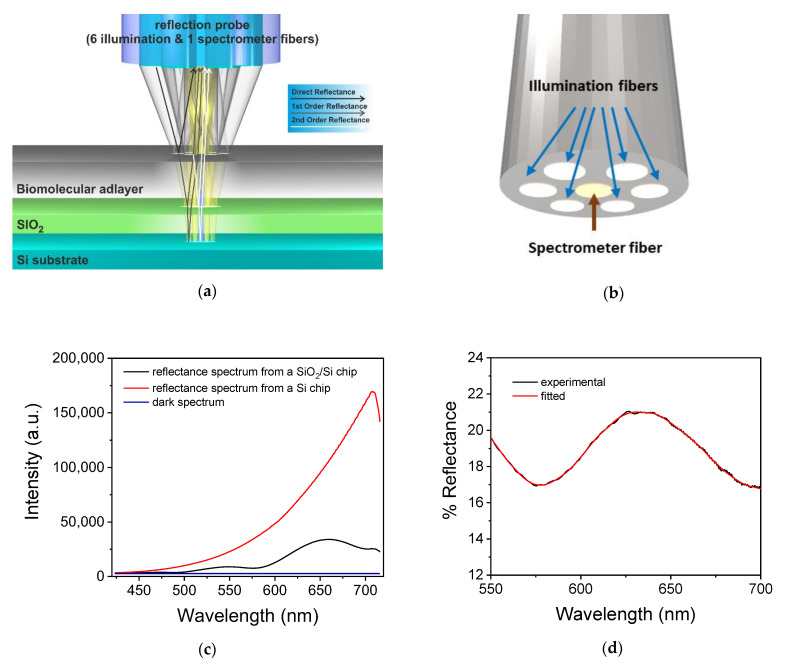
(**a**) Schematic of WLRS detection principle showing the paths of the light from the six illumination fibers of the reflection probe and the collection of the reflected light by the central spectrometer fiber. The light is reflected by the Si substrate (2nd order reflectance), then at the Si/SiO_2_ and SiO_2_/biomolecular adlayer interface (1st order reflectance), and finally by the biomolecular adlayer/solvent interface (direct reflectance), creating an interference spectrum. Reprinted with permission from [94] copyright (2017) Elsevier Ltd. (**b**) Schematic of the reflection probe showing the positioning of the six illumination fibers at its periphery and of the seventh central fiber that collects the reflected light and guides it to the spectrometer. (**c**) Graph showing the reference reflectance spectrum acquired from a Si chip (red line), the dark spectrum acquired with the light source turned off (blue line), and the reflectance spectrum SiO_2_/Si chip (black line). (**d**) Graph presenting a typical normalized experimental reflectance spectrum (black line) and the respective fitted spectrum (red line).

In its current form, the WLRS immunosensing platform employs a Si chip with a transparent SiO_2_ layer on it, which operates as a sensing surface through immobilization of a suitable molecule, e.g., a primary or secondary antibody or a suitable analyte–protein bioconjugate. The biofunctionalized Si chip is coupled with a tailor-made microfluidic cell through which the assay solutions are continuously delivered, forming a biochip. The reader of the WLRS biochip includes a stabilized broadband light source, a high resolution in terms of both intensity and spectral content spectrometer, and a dedicated reflection probe consisting of seven optical fibers: six at the periphery of the probe, which direct the light from the source to the biochip surface, and one at the center of the probe, which collects the light reflected by the biochip and directs it to the spectrometer. The light passes through the transparent microfluidic cell and is directed to the sensing surface vertically, and is reflected at the various interfaces (the sample under analysis/functionalized layer, biofunctionalized layer/SiO_2_ layer, and SiO_2_ layer/Si substrate) due to the difference in the refractive index between adjacent layers (Figure 1a). Thus, interference occurs at each wavelength, resulting in an interference spectrum which can be collected by the central fiber of the reflection probe (Figure 1b). The increase in thickness of the biomolecular adlayer caused by the biomolecular interactions on the sensing surface leads to red-shifting of the interference spectrum. The WLRS reader is combined with specially developed software which can evaluate the initial thickness of the SiO_2_/biomolecular adlayer and transform the shift of the interference spectrum into the effective thickness of the biomolecular adlayer (nm), which is actually the signal of the WLRS sensor. More specifically, a reference [Ref(λ)] and a dark spectrum [D(λ)] are obtained prior to real-time continuous recording of the reflectance spectrum [S(λ)] (Figure 1c), and the absolute reflectance spectrum is calculated by Equation (1):

R(λ) = S(λ) − D(λ)/(Ref(λ) − D(λ))(1)

The normalized spectrum is further processed through the Levenberg–Marquart algorithm [70] to calculate the thickness of the biomolecular adlayer (Figure 1d), d_1_, from the shift in the interference spectrum wavelength, δλ, according to Equation (2):

Δλ = r1 × [1 − r_2_^2^/(r_1_ + r_2_) × (1 + r_1_ × r_2_)] × (n_1_ × d_1_/n_2_ × d_2_) × λ_0m_ (2)

where r_1_ and r_2_ and n_1_ and n_2_ are the Fresnel coefficients and refractive indices of the biomolecular and the SiO_2_ layer, respectively, d_1_ and d_2_ are the thickness of the two layers, and λ is the wavelength, where λ_0m_ is the reflectance extremum.

In this format, the WLRS-sensing platform is suitable for label-free real-time monitoring of the biomolecular interactions taking place on the Si/SiO_2_ chip, with a detectable change in effective adlayer thickness < 0.1 nm.

**Figure 2:**
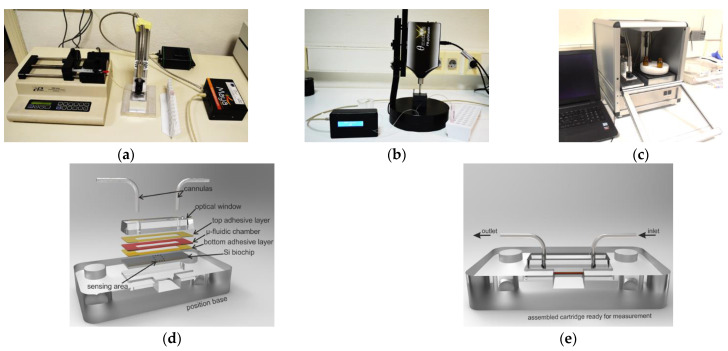
(**a**–**c**) WLRS instrumentation formats that have been developed over the years, starting from an off-the-shelf format (**a**) to more integrated ones (**b**,**c**). (**d**) Main parts of the microfluidic module. (**e**) Chip assembled with the microfluidic module and placed on the docking station.

It should be noted that various improvements have been incorporated into WLRS-immunosensing devices over the years (Figure 2). As an example, a single channel spectrometer was introduced [90] to replace the beam splitter and double spectrometer previously employed to receive the reference and reflectance spectrum at the same time [91]. The most recent improvements include the following: integration of all optical and electronic components, along with the peristaltic pump, a computer-controlled carousel for the reagents’ solutions, and a sampler into a compact instrument of rather small size (L × W × H 32 × 36 × 38 cm); stabilization of the light source to allow for long-term operation; selection of spectral range concerning the spectrometer employed; and enrichment of the software to enhance automation from assay performance up to data acquisition.

The first bioanalytical applications of the WLRS sensing platform [91,92,93,94,95,96] were previously described in [89]. Both these and later-developed optical immunosensors based on White Light Reflectance Spectroscopy have been included in Table 2, which summarizes the critical features of the sensors developed.

All WLRS immunosensors employ proper biochips. More specifically, Si chips (5 × 15 mm^2^) covered with a thin (~1000 nm) SiO_2_ layer were suitably treated with O_2_ plasma or Piranha solution and subsequently with 2%, *v*/*v*, aqueous 3-aminopropyl triethoxysilane (APTES) solution, as previously described in detail [86,93], while biofunctionalization of the chip surface was achieved through immobilization of a suitable biomolecule depending on the immunoassay format/design, either by adsorption or covalent binding. Biofunctionalization of distinct areas of the surface with different biomolecules has led to the development of multiplexed-type immunosensors [93,94,97]. On the other hand, as described in a recent application, 3D structuring of areas with different thickness on the SiO_2_ layer, which were biofunctionalized with different biomolecules and then analyzed with a single reflection probe (Multi Area Reflecting Spectroscopy, MARS) and suitable software, has led to a multiplexed immunosensor as well [98]. In all cases, the WLRS-sensors were based on the immobilization of a suitable biomolecule, e.g., a primary antibody, on the Si/SiO_2_ chip, with no need to use any type additional specific materials, which are often developed in-house and can be expensive.

A variety of antibodies (mammalian IgGs or avian IgYs, monoclonal or polyclonal, in-house developed or commercially available) and assay formats (competitive and non-competitive) have been employed by the WLRS platform. Concerning primary antibodies, it is worth mentioning rabbit polyclonal ones recognizing benzimidazole-type pesticides, which have been developed in-house starting with low-cost commercially available chemicals. These antibodies, which were preliminarily integrated into a cell-based electrochemical sensor [99,100], have proven well suited to the WLRS platform and been applied to the determination of carbendazim in fruit juices [86]. Moreover, biotinylated antibodies, either primary or secondary, were often used in the WLRS platform in combination with streptavidin, thus taking advantage of the well-established advantages of the (strept)avidin–biotin system [101] to increase sensitivity and improve the detection limit of the immunosensor.

In all cases, the WLRS-based immunosensors exhibited very good analytical characteristics, i.e., high sensitivity and precision, along with the practical advantages of high economic impact, such as short analysis time and re-usage ability. Thus, almost all of the WLRS immunosensors developed to date have been extensively tested for their ability to perform multiple sequential analyses onto a single biochip. All tested sensors exhibited considerably high stability, with regeneration cycles ranging from 12 up to 30 (Table 2). Particular attention has been paid to keeping total analysis time as short as possible through optimization studies; thus, in most cases the time needed to analyze a sample was less than 30 min, while direct-type assays with analysis time as short as 60 sec have been described [90,91].

The WLRS platform has been well suited to the immunodetection of various entities, ranging from whole microorganisms, i.e., bacteria cells [88] and high molecular weight biomolecules [87,90,91,92,93,95,96], to low molecular weight compounds [85,86,94,97,102,103,104], which proves the high versatility and wide applicability of WLRS immunosensors. Consequently, the WLRS immunosensors developed thus far have been applied to various areas of interest and various types of samples, from disease diagnosis (determination of C-reactive protein and/or D-dimer as inflammation biomarkers in human serum/whole blood, detection of the complement activation product C3b and its metabolites in human serum as autoimmunity indicators) [87,91,93,96] and determination of total and free PSA as cancer biomarkers [92] to food analysis and monitoring of environmental pollution (detection of *Salmonella typhimurium* in water samples [88], determination of the natural toxins, ochratoxin A in flour [85], deoxynivalenol in cereals [102], aflatoxin B1 and fumonisin B1 in wheat and maize [98], and aflatoxin M1 in milk [104], and the determination of the pesticides carbendazim in fruit juices [86], glyphosate in drinking water [103], paraquat and atrazine in water [97], and chlorpyrifos, imazalil, and thiabendazole in water and wine [94]). Moreover, a WLRS-based immunosensor has been developed and applied to the field of forensic sciences as well [95] (determination of PSA as semen indicator).

## 4. Discussion and Future Perspectives

Recent research efforts in the field of optical immunosensors have mainly focused on two directions. First, the improvement of the analytical assay characteristics, e.g., sensitivity and specificity, which are predominantly, although not exclusively, dependent on the biomolecules used for biorecognition (in our case, anti-analyte antibodies) along with the overall assay format/design. Second, efforts have been directed towards building up transduction systems of improved characteristics, such as fluorescence-quenching systems with high sensitivity/low background at the detection level; moreover, intense effort has been invested in the development of label-free systems, such as SPR- and WLRS-based platforms, with high analytical performance, high versatility, robustness, low cost, and the ability to be used at the Point-of-Care. Research on signal enhancement systems is a topic of continuous interest and high importance, although it has been considered outside the main scope of this article.

Concerning efforts to improve biorecognition in optical immunosensors, tailor-made antibodies with desirable characteristics have been employed in several of the most recently reported optical immunosensing platforms (Table 1 and Table 2), although commercially available mouse monoclonals remain the antibodies most widely used. Alternative approaches include antibodies isolated from the egg yolk of avian species (IgYs). Avian antibodies are considered to offer better specificity and overall efficiency in assays targeting biomolecules of mammalian origin due to higher phylogenetic distance between mammalian and avian species [70,105]. Other features of avian IgYs, such as higher molecular weight in comparison with that of their mammalian counterparts (180 kDa vs. 150 kDa), may prove to be advantageous for application to certain types of sensors, e.g., label-free reflectrometric immunosensors, in which the thickness of the analyte–antibody adlayer is of critical importance. Other qualities of avian IgYs, such as high robustness and low cost, as well as avoidance of animal bleeding, which offers better compliance with the “3Rs” ethical principle governing research with animals (Replacement—Reduction—Refinement) [68,106], may help IgYs to become more widely applied as biorecognition molecules in optical immunosensors. Moreover, in accordance with the recommendation of the European Union Reference Laboratory for Alternatives to Animal Testing (EURL ECVAM) [107], non-animal-derived antibodies might be eventually applied to immunosensors along with other products of synthetic biology, such as aptamers or molecular imprinted polymers [108,109], provided that current problems related to high cost, low availability, and often poor analytical features of the latter can be solved. Alternatively, combined use of totally-synthetic biorecognition molecules and traditional antibodies might prevail [110,111]. Last but not least, it should be noted that chemical functionalization of sensing surfaces so as to enable/facilitate efficient immobilization and better orientation of antibodies or other suitable biomolecules onto the sensing surface is an issue of special interest.

Concerning the development/improvement of label-free transducer systems, several relevant recent research efforts have been presented in this work (Table 1 and Table 2). Among label-free optical immunosensors, the WLRS-based sensors (Table 2) show a series of advantageous inherent features, such as simple and robust instrumentation, use of inexpensive sensing material(s) (i.e., Si/SiO_2_), low interference from sample matrix, and high versatility concerning suitable analytes (ranging from whole bacteria cells and high molecular weight disease biomarkers to low molecular weight food toxins). The above advantages, along with the high potential of adopting/integrating further improvements, especially toward construction of a small-size, low weight, ideal for point-of-need applications, “all-in-one” functional device, may eventually render the WLRS-based immunosensing platform an invaluable analytical tool with high applicability.

## 5. Conclusions

New optical immunosensors are continuously being developed in an attempt to eventually achieve the construction of an “ideal” bioanalytical device, overcoming current problems and pitfalls. In this review paper, new developments in the field have been presented, including recent bioanalytical applications of optical immunosensors, while future perspectives have been briefly discussed. Special focus has been directed to the label-free WLRS-based immunosensing platform, which is considered a very promising tool for conducting bioanalytical studies in many areas of interest.

## Figures and Tables

**Table 1:** Reports on optical immunosensors published in 2021–2022.

Labeled/ Label-Free	Optical Signal/Method	Antibodies (Abs) and Signal-Sensitive Material(s) Employed	Immunoassay Format	Analyte/Application Area	Ref.
Labeled	Fluorescence	Commercially available monoclonal Abs immobilized on graphene quantum dots (energy donor) along with Ti_3_C_2_-MXene decorated with silver nanoparticles (energy acceptor)	non-competitive (direct-type) ^a^	Neuron Specific Enolase/Health	[56]
Labeled	Fluorescence	Commercially available Abs immobilized on carbon dots and silver nanoparticles	non-competitive (sandwich-type) ^b^	Human Epididymis Protein 4 and/or ovarian cancer cells/Health	[57]
Labeled	Fluorescence (FRET-based)	Commercially available Abs immobilized on iron porphyrin bio-mimicking graphene quantum dots	non-competitive (direct-type)	*Salmonella typhi VI* antigen in human serum/Health	[59]
Labeled	Fluorescence	In-house developed mouse monoclonal Abs immobilized through protein A onto cell-based fluorescent microspheres containing carbon dots	non-competitive (direct-type)	*Escherichia coli* O157:H7 in milk/Food Analysis	[60]
Labeled	Fluorescence	Commercially available mouse monoclonal Absimmobilized on quantum dot nanobeads	competitive	Aflatoxin B1 in lotus seeds/Food Analysis	[58]
Labeled	Fluorescence (Evanescent-wave-based)	Commercially available Abs labeled with Cy5.5 along with a light-sheet skew rays enhanced U-shaped optical fiber	competitive	Microcystin-LR in drinking and lake water/Food Analysis & Environmental Pollution Monitoring	[61]
Labeled	Fluorescence	Antibodies indirectly immobilized on a glass fiber surface and used as an antibody-analyte-aptamer structure along with a CRISPR/Cas12a fluorescence detection system	non-competitive (sandwich-type)	Small proteins of biomedical interest, e.g., INF-γ in human serum, whole blood, perspiration and saliva samples/Health	[62]
Labeled	Fluorescence/Colorimetry	Monoclonal Abs coupled to *Staphylococcus aureus*-biosynthesized polymer dots, which can generate colorimetric-fluorescent dual signals	non-competitive (direct-type)	Zearalenone in millet and corn samples/Food analysis	[55]
Labeled	Phosphorescence (quenching-based)	Commercially available rabbit polyclonal Abs along with a phosphorescent tracer, i.e., analyte labeled with palladium-tetrabenzoporphyrin	competitive	Estrone and Estradiol/Health & Environmental Pollution Monitoring	[63]
Labeled	Chemiluminescence	Commercially available biotinylated Abs combined with streptavidin-biotinylated HRP nanocomposites and a HRP/chemiluminescent substrate; an optical fiber was used for signal guidance, on which an analyte-protein bioconjugate was immobilized	competitive	Methamphetamine in human blood, urine and oral fluid/Forensic Analysis	[64]
Labeled	Chemiluminescence	Commercially available mouse monoclonal Abs labeled with HRP along with a proper chemiluminescent substrate embedded on glass fiber pad of a sensor-strip	non-competitive (direct-type)	IgAs against proteins of SARS-CoV-2 in human serum and saliva/Health	[65]
	Colorimetry	Commercially available mouse monoclonal Abs immobilized onto gold nanoparticles	non-competitive (direct-type)		
Labeled	Colorimetry	Commercially available Abs Immobilized onto cotton swabs (capture Abs) and blue-colored polymeric nanobeads (detection Abs)	non-competitive (sandwich-type)	Methicillin resistant *Staphylococcus aureus* (MRSA)/Health	[54]
Label-free	Dynamic light scattering (DLS)	Commercially available rabbit polyclonal Abs immobilized onto gold nanoparticles	non-competitive (direct-type)	SARS-CoV-2 virus/Health	[66]
Label-free	Scattering of evanescence light	Commercially available monoclonal Abs conjugated to light scattering particles (capture Abs)/immobilized on the surface of optical waveguide film (detection Abs)	non-competitive (sandwich-type)	Nucleocapsid protein of SARS-CoV-2 virus/Health	[71]
Label-free	Retroreflectometry	Commercially available mouse monoclonal Abs immobilized on PMMA-biosensing chips (capture Abs)/conjugated to retroreflective microparticles (detection Abs)	non-competitive (sandwich-type)	Creatine kinase-myocardial band in buffer and spiked human serum/Health	[80]
Label-free	Fabry–Pérot interferometry (FPI)	Commercially available Abs immobilized on parylene-C nanosheet/Si	non-competitive (direct-type)	Prostate Specific Antigen/Health	[81]
Label-free	Polarization interferometry	Commercially available polyclonal Abs immobilized on a waveguide composed of Si_3_N_4_/SiO_2_ on Si	non-competitive (direct-type)	*Fusarium* mycotoxin Zearalenone in buffer/Food Analysis	[82]
Label-free	Refractometry	In-house developed avian (hen) polyclonal Abs (IgYs) immobilized on the grating region of a long-period fiber grating (LPFG) sensor	non-competitive (direct-type)	*Staphylococcus aureus* in water samples/Food Analysis	[68]
Label-free	Refractometry	Commercially available goat polyclonal Abs immobilized on the surface of an elliptical core helical intermediate-period fiber grating (E-HIPFG) sensor	non-competitive (direct-type)	Human immunoglobulin G/Health	[69]
Label-free	Refractometry	Commercially available monoclonal Abs immobilized on the surface of a Microfiber Bragg grating sensor	non-competitive (direct-type)	Cardiac Troponin I (cTn-I)/Health	[78]
Label-free	Photoluminescence	Commercially available monoclonal Abs immobilized on polyacrylonitrile/zinc oxide (PAN/ZnO) nanofibers	non-competitive (direct-type)	Aflatoxin B1 in buffer/Food Analysis	[67]
Label-free	SPR	Commercially available Abs immobilized on the surface of a gold chip -along with an aptamer conjugated to magnetite nanorods containing ordered mesocages and silver nanoclusters (MNOM@AgNCs)	non-competitive (sandwich-type)	Programmed death ligand 1 (PD-L1) in human plasma/Health	[73]
Label-free	SPR	Commercially available polyclonal Absimmobilized onto a gold/palladium coated unclad plastic optical fiber	non-competitive (direct-type)	Cortisol in buffer/Health and/or Food Analysis	[74]
Label-free	SPR	Tailor-made rabbit (IgGs) and avian (IgYs) polyclonal Abs immobilized on gold-coated glass surface	non-competitive (direct-type)	Hazelnut allergen Cor a14/Food Analysis	[70]
Label-free	SPR	Enzymatically half-reduced mouse monoclonal Abs immobilized on self-assembled gold nanoparticles	non-competitive (direct-type)	Collagen I/Health	[72]
Label-free	SPR	Mouse (and humanized) monoclonal Abs immobilized on the chip surface through protein G	non-competitive (sandwich-type)	Ricin, abrin biotoxins in human plasma as well as crude extracts of food samples/Health and/or Food Analysis	[75]
Label-free	SPR	Pair of mouse monoclonal Abs, immobilized on the gold sensing surface (capture antibodies) or coupled onto gold-coated magnetic nanoparticles (detection antibodies) leading to signal amplification	non-competitive (sandwich-type)	CD5 in spiked human sera/Health	[76]
Label-free	Surface Enhanced Raman Scattering (SERS)	Commercially available Abs immobilized on Fe_3_O_4_ magnetic nanoparticles (for signal enhancement) along with analyte labeled with a Raman-tag and gold (core)/silver(shell) bimetallic nanocuboid (Raman probe/tracer)	competitive	Imidacloprid in water /Environmental Pollution Monitoring	[77]
Label-free	Optical Waveguide Lightmode Spectroscopy (OWLS)	In-house developed rabbit polyclonal Abs either directly (a) or indirectly, i.e., through an analyte-bioconjugate (b) immobilized on SiO_2_-TiO_2_ chips	(a) non-competitive (direct-type); (b) competitive	*Fusarium* mycotoxin zearalenone in maize/Food analysis	[79]
Label-free	Light Diffraction	Abs against the SARS-CoV-2 spike protein immobilized on a polymer-type imprinted photonic crystal film	non-competitive (direct-type)	SARS-CoV-2 spike protein/Health	[83]
Label-free	Total internal reflection ellipsometry	Recombinant spike proteins of three SARS-CoV-2 variants (wild type, B.1.1.7. and B.1.351) immobilized on gold-coated SPR sensor disc	non-competitive (direct-type)	Abs circulating in human sera after vaccination with the Vaxzevria vaccine which could recognize the spike proteins of three SARS-CoV-2 variants/Health	[84]
Label-free	White light reflectance spectroscopy (WLRS)	In-house developed rabbit polyclonal Abs immobilized through an analyte-bioconjugate onto a Si/SiO_2_ chip	competitive	Ochratoxin A in cereal flours/Food Analysis	[85]
Label-free	WLRS	In-house developed rabbit polyclonal Abs immobilized through an analyte-bioconjugate onto a Si/SiO_2_ chip	competitive	Carbendazim in fruit juices/Food Analysis	[86]
Label-free	WLRS	Commercially available rabbit polyclonal Abs immobilized through *Salmonella* LPS onto a Si/SiO_2_ chip	competitive	*Salmonella typhimurium* in drinking water/Food Analysis	[88]
Label-free	WLRS	Commercially available goat polyclonal Abs immobilized onto a Si/SiO_2_ chip (capture Abs); the same Abs were used for detection	non-competitive (sandwich-type)	C-Reactive Protein in human blood/Health	[87]

^a^: non-competitive format (direct-type): In this, one primary anti-analyte antibody is used that is usually immobilized on the transducer surface and the binding of the analyte is directly recorded. ^b^: non-competitive format (sandwich-type): In this, a pair of primary anti-analyte antibodies is used, consisting of the so-called capture and detection antibodies. The capture antibodies are usually immobilized on the transducer surface and bind the analyte, while the detection antibodies, which are often appropriately labeled, bind to the analyte molecule through a different epitope before the “sandwich-type” binding of the analyte is finally recorded.

**Table 2:** Reports on WLRS-based immunosensors published in 2009–2022.

Analyte	Functionalizing Biomolecule	Type of Antibodies (Abs)	Immunoassay Format	Signal Enhancement	Matrix/ Sample	Analysis Time	Regeneration Cycles	Refs
Ochratoxin A (OTA)	OTA-Ovalbumin (OTA-OVA) conjugate	In-house developed rabbit polyclonal Abs	Competitive	Yes (Biotinylated secondary ^a^ Ab/streptavidin)	Cereal flours	25 min	N/A	[85]
Carbendazim	Benzimidazole derivative-Oligolysine conjugate	In-house developed rabbit polyclonal Abs	Competitive	Yes (Biotinylated secondary Ab/streptavidin)	Fruit juices	28 min	12	[86]
*Salmonella typhimurium* (bacteria cells)	*Salmonella* LPS	Commercially available rabbit polyclonal Abs	Competitive	Yes (Biotinylated secondary Ab/streptavidin)	Water	15 min	15	[88]
C-reactive protein (CRP)	anti-CRP Abs	Commercially available goat polyclonal Abs for capture and detection	Non-competitive (sandwich-type)	No	Human plasma	12 min	N/A	[87]
Deoxynivalenol (DON)	DON-OVA conjugate	Commercially availabl emouse monoclonal Abs	Competitive	Yes (Secondary Ab)	Cereals	12 min	20	[102]
Aflatoxin B1 (AFB1) and Fumonisin B1 (FB1)	AFB1-Bovine Serum Albumin (AFB1-BSA) & FB1-OVA conjugates	Commercially available mouse monoclonal Abs	Competitive	Yes (Secondary Ab)	Wheat and maize	12 min	N/A	[98]
Glyphosate	Glyphosate-BSA conjugate	Commercially available avian polyclonal Abs (IgYs)	Competitive	Yes (Secondary Ab)	Water	25 min	14	[103]
Atrazine and Paraquat	Atrazine-BSA & Paraquat-BSA conjugates	Commercially available rabbit polyclonal anti-paraquat Abs and mouse monoclonal anti-atrazine Abs	Competitive	Yes (Secondary Ab)	Water	12 min	20	[97]
Aflatoxin M1 (AFM1)	AFM1-BSA conjugate	Commercially available rabbit polyclonal Abs	Competitive	Yes (Biotinylated secondary Ab/streptavidin)	Milk	25 min	25	[104]
C-Reactive Protein (CRP)	anti-CRP Abs	Commercially available goat polyclonal Abs for capture and detection	Non-competitive (sandwich-type)	Yes (Biotinylated detection Ab/str)	Whole blood	12 min	N/A	[96]
Prostate Specific Antigen (PSA)	anti-PSA Abs	Commercially available goat polyclonal Abs for capture and detection	Non-competitive (sandwich-type)	Yes (Biotinylated detection Ab/streptavidin)	Forensic samples/semen samples	10 min	24	[95]
Chlorpyrifos, Imazalil and Thiabendazole	Chlorpyrifos-BSA, Imazalil-BSA & Thiabendazole-BSA conjugates	Commercially available monoclonal Abs	Competitive	Yes (Secondary Ab)	Water, wine	10 min	30	[94]
CRP and D-dimer	anti-CRP Abs	Commercially available goat polyclonal Abs for capture and detection.	Non-competitive (sandwich-type)	No	Human plasma	45 min	25	[93]
	anti-D dimer Abs	Commercially available goat polyclonal Abs for capture and mouse monoclonal Abs for detection	Non-competitive (sandwich-type)	Yes (Biotinylated detection Ab/streptavidin)				
PSA (total and free)	anti-totalPSA & anti-freePSA Abs	Commercially available goat polyclonal anti-total PSA Abs for capture and detection and a pair of mouse monoclonal anti-freePSA Abs	Non-competitive (sandwich-type)	Yes (Biotinylated detection Ab/streptavidin)	Human serum	65 min	20	[92]
Mouse IgG	anti-mouse IgG Abs	Commercially available goat polyclonal Abs	Non-competitive (direct-type)	No	Buffer	1 min	7	[90]
Complement Activation Products (C3b)	anti-C3b Abs	Tailor-made mouse monoclonal Abs	Non-competitive (direct-type)	No	Human serum	1 min	N/A	[91]

^a^: Secondary Ab: anti-species antibody recognizing the IgG of the host animal, i.e., the animal in which the primary anti-analyte antibody has been developed.

## Data Availability

Not applicable.
